# Prevalence and predictors of HIV testing among young men in Papua New Guinea: A cross-sectional analysis of a nationally representative sample

**DOI:** 10.1371/journal.pone.0306807

**Published:** 2024-08-14

**Authors:** McKenzie Maviso

**Affiliations:** Division of Public Health, School of Medicine and Health Sciences, University of Papua New Guinea, Port Moresby, Papua New Guinea; Torrens University Australia, AUSTRALIA

## Abstract

**Background:**

HIV testing is an important component of HIV prevention and serves as a gateway to other HIV-related services. However, the uptake remains suboptimal among young people, particularly in highly prevalent settings such as Papua New Guinea (PNG). This study aimed to assess the prevalence and determine the predictors of HIV testing uptake among young men aged 15–24 years in PNG.

**Methods:**

The 2016–2018 PNG Demographic and Health Survey (DHS) data was used. A total of 1,275 young men aged 15–24 years were included in the final analysis. Descriptive, bivariate, and multivariable logistic regression analyses were performed to determine independent predictors of HIV testing. Adjusted odds ratios (AORs) with 95% confidence intervals (CIs) were reported. All analyses were adjusted using survey weights to account for unequal sampling probabilities.

**Results:**

The overall prevalence of HIV testing among young men was 17.1% (95% CI: 15–19). Of those who were tested for HIV, about one-third (32.9%) had experienced a sexual debut at age <15 years, and 33.9% inconsistently used condoms during sex. In multivariable analysis, men aged 20–24 years (AOR 1.18, 95% CI: 1.00–2.31), who owned mobile phones (AOR 1.43, 95% CI: 1.00–2.55), who were aware that consistent condom use during sex can reduce HIV risk (AOR 2.18, 95% CI: 1.18–4.04), who had paid for sex (AOR 1.75, 95% CI: 1.01–5.83), and who had two or more sexual partners (AOR 1.37, 95% CI: 1.01–3.14) had increased odds of HIV testing. However, decreased odds of HIV testing were found among men who were never married (AOR 0.51, 95% CI: 0.29–0.88), lived in rural areas (AOR 0.54, 95% CI: 0.32–0.92), and consistently used condoms during sex (AOR 0.59, 95% CI: 0.34–1.01).

**Conclusion:**

The findings show that HIV testing is low among young men in PNG. To increase HIV testing uptake among young men, it is crucial to implement comprehensive youth-friendly HIV/STI education and tailored sensitization programs and enable more accessible and affordable HIV testing services. Also, outreach and community-based testing programs for young men in rural and prioritized areas requiring urgent prevention interventions are feasible options in PNG.

## Introduction

Considerable progress has been made in responding to HIV/AIDS under the Sustainable Development Goals framework to end the epidemic by 2030 [1]. Since 2010, new HIV infection rates have decreased by 14%, and 78% of people living with HIV know their status [2]. However, in the Asia and Pacific region, the HIV response remains a challenge and is impacted by marked disparities and varied epidemic trends, notably affecting young people aged 15–24 years, who represent a growing proportion of people disproportionately affected by the burden of HIV [2]. In 2022, an estimated 440,000 young people were living with HIV, accounting for about 25% of new HIV infections in the region [2].

The United Nations’ 95-95-95 targets to end the HIV epidemic by 2030 [3]. One of the targets called for 95% of people living with HIV to know about their serostatus [3, 4]. HIV testing is a key strategic entry point to prevention, treatment, care, and support services. In addition, HIV testing confers multiple benefits for individuals who test positive and those who test negative and encourages preventive behaviors [5, 6]. Despite the significant benefits, young people remain the least likely to get an HIV test and know their HIV status [7, 8]. Studies have shown that not all, particularly men willingly seek HIV testing services as a way to prevent HIV transmission and/or acquisition due to various barriers, such as individual (poor HIV/AIDS knowledge, testing availability, and unwillingness to test), social (stigma, discrimination, and lack of social support), and health service utilization (loss of trust or confidence in health workers and lack of medication, including ART) [9–11]. These barriers undermine efforts to increase HIV testing and highlight the critical need to develop appropriate HIV prevention strategies.

Among young people, their sexual behaviors and susceptibility to HIV infection differ widely by social context, as well as increased individual autonomy and a lack of social control [12, 13]. For example, high-risk behaviors such as early sexual debut, substance use (alcohol consumption and drug use), inconsistent condom use, and multiple and concurrent sexual relationships, as well as poor HIV knowledge, low-risk perception, and sensation-seeking behaviors, increase HIV risks and transmissions among young people [14–17]. In addition, biological factors such as low rates of male circumcision [18, 19] and manifestations of other sexually transmitted infections (STIs) (chlamydia, gonorrhea, syphilis, and trichomoniasis) [20, 21] contribute to some of the increased HIV risk and burden among this population. Despite young people’s vulnerability to HIV infection, they are frequently overlooked in the design and implementation of national HIV/AIDS prevention strategies [22, 23]. They remain an important target group for HIV prevention and STI surveillance due to their unique behavioral and social-related vulnerability.

PNG is one of the culturally diverse countries in the Western Pacific region and is grappling with a concentrated-generalized HIV epidemic, indicating significant disparities in HIV prevalence between key populations (e.g., sex workers and men who have sex with men) and the general population [24, 25]. HIV prevalence is 1% in the general adult population [26]. Among young people, the prevalence of HIV is estimated at 0.2% for males and 0.5% for females [26], demonstrating the high vulnerability of this priority group in the country. Although HIV testing services are widely available throughout PNG [27], the current testing rate among young people is suboptimal, especially among those most at risk of HIV [28, 29]. Furthermore, a major obstacle in the country continues to be young men’s participation in HIV programs and their access to sexual and reproductive health care [30, 31]. Evidence concerning HIV knowledge and risk reduction behaviors (e.g., consistent use of condoms and having only one faithful sexual partner) among young men is scarce; although a recent study among young women in PNG found that early sexual debut, having one sexual partner and not having an STI were associated with not testing for HIV [28]. However, young men’s knowledge of HIV and sexual relationships is established through a complex interplay of sociocultural norms, expectations, attitudes, and information gleaned from various sources [32].

Despite the established profile of risky sexual behaviors among young people [28, 33, 34], little is known about the prevalence and correlates of HIV testing uptake in young men in PNG. Moreover, evidence concerning predictors influencing the uptake of HIV testing among young men is scarce. This creates a critical knowledge gap that must be filled to inform national-level policy development and targeted interventions. In this study, data from a nationally representative survey was used to estimate the prevalence and predictors of HIV testing uptake among young men aged 15–24 years in PNG.

## Methods and materials

### Data sources

Data for the current study were derived from the 2016–2018 PNG Demographic and Health Survey (DHS), which used a stratified, multistage cluster sampling method to ensure a sample representative of the population. The survey was conducted between October 2016 and December 2018 and collected data on the country’s important demographic, socioeconomic, and health indicators. The survey used the list of census units (CUs) from the 2011 PNG National Population and Housing Census [35] as the sampling frame, which contains information on CU location, type of residence (urban or rural), estimated number of residential households, and population by sex. Administratively, the country is divided into four (4) main regions (Southern, Highlands, Momase, and Islands) comprising 22 provinces, and each province is subdivided into urban and rural areas. The PNG National Statistical Office (NSO) with technical support and assistance from the Inner City Fund (ICF) through the DHS Program conducted the study. The governments of PNG and Australia, the United Nations Population Fund (UNFPA), and the Children’s Fund of the United Nations (UNICEF) funded the survey [36].

### Sampling procedures

The 2016–2018 PNGDHS sample stratification and selection were achieved in two strata: urban and rural areas, except for the National Capital District, which has no rural areas. In each stratum, samples of households were selected in two stages, with CU constituting the primary sampling unit and households as the secondary sampling unit. The first stage involved selecting 800 CUs using a probability proportional to the CU size. In the second stage, 24 households were selected from each cluster using probability sampling, yielding a total sample size of approximately 19,200 households. A total of 7,333 men aged 15–49 years participated in the survey. Young men who never had sex and aged 25 or more were excluded from this study. A total weighted sample of 1,275 young men aged 15–24 years who ever had sex with variables of interest were included in this study. Details about the sampling technique, survey team training, household selection, survey questionnaires, and validation procedures are available in the final report [36].

### Variables and measurements

The primary outcome variable of this study was “ever tested for HIV?” and was dichotomized as “0” for “no” or “1” for “yes.” The explanatory variables were selected based on the literature and their relevance [18–20] and were divided into three categories: (1) Sociodemographic factors include age, marital status, educational level, employment status, wealth index, region, and place of residence. Also, household factors measured included exposure to mass media (listening to the radio and watching television), access to the internet, and mobile phone ownership. (2) HIV-related knowledge includes ever heard of AIDS or STIs, can a healthy person have HIV? Can a person get HIV by sharing food with an infected person? Can a person get HIV through witchcraft? Consistent condom use can reduce HIV risk and knowing an HIV testing facility. (3) Sexual risk behaviors include age of sexual debut, number of sexual partners (including spouse), having ever paid for sex, and consistent condom use during sex, and having had an STI (unspecific) in the last 12 months.

### Statistical analysis

Data extraction, recoding, and analysis were performed using IBM Statistical Package for the Social Sciences (SPSS), Version 26.0 (Armonk, NY: IBM Corp.). The sample was weighted using the primary sampling unit variable, stratification variable, and weight variable to restore its representativeness and obtain a better estimate throughout the analysis. Descriptive statistics were used to explore the characteristics of the sample and were presented as weighted frequencies (*n*) and percentages (%). A chi-square test for independence was performed to assess the strength of the association between the dependent variable and each independent variable. Variables that were significant and those with *p* ≤ 0.25 in the bivariate analysis were included in a multivariable analysis to determine their collective associations with HIV testing [37]. Since the PNGDHS used a two-stage stratified sampling technique, a complex samples analysis technique was employed to determine independent predictors of HIV testing. Adjusted odds ratios (AORs) with 95% confidence intervals (CIs) were reported. A *p* ≤ 0.05 was used to assess statistical significance.

### Ethical considerations

Permission to use data was obtained from the DHS program (https://dhsprogram.com) and was only used for this study. The Institutional Review Board (IRB) of Inner City Fund (ICF) International and the IRB committee of PNG examined and approved the 2016–2018 PNGDHS protocols. All the respondents had provided verbal informed consent before each interview. There were no ethical considerations on the researcher’s part as the data were anonymized entirely, with no identifiable information on the survey participants when analyzed.

## Results

### Sociodemographic characteristics of young men

Table 1 presents the participants’ characteristics. Overall, 1,275 sexually active young men aged 15–24 years enrolled in the study. The mean age was 20.84 (± 2.31) years, while the majority (70.2%) were aged 20–24 years and lived in rural areas (82%). Nearly half had attained primary (44.3%) and secondary (45.5%) education, and about two-thirds (62%) were unemployed. Regarding mass media exposure, more than two-thirds of young men listened to the radio (71.7%), while less than half watched television (47.1%) and had internet access (33.6%). More than half (57.8%) of young men owned mobile phones.

**Table 1:** Sociodemographic characteristics of participants (N = 1,275).

Characteristics	Frequency	
	*n*	%
***Sociodemographic***		
Age (years)		
15–19	380	29.8
20–24	895	70.2
Mean age = 20.8 (± 2.31) years		
Marital status (missing, *n* = 26)		
Never married	940	75.2
Married	310	24.8
Educational level		
No formal education	73	5.7
Primary	565	44.3
Secondary	580	45.5
Tertiary	58	4.5
Employment status		
Not employed	791	62.0
Employed	485	38.0
Wealth Index		
Poorest	120	9.4
Poorer	119	9.3
Middle	189	14.8
Richer	294	23.0
Richest	553	43.4
Place of residence		
Urban	229	18.0
Rural	1,046	82.0
Region		
Southern	300	23.6
Highlands	455	35.7
Momase	373	29.2
Islands	147	11.5
Read newspaper/magazine		
No	369	28.9
Yes	907	71.1
Listen to the radio		
No	361	28.3
Yes	914	71.7
Watch television		
No	674	52.9
Yes	601	47.1
Internet access		
No	847	66.4
Yes	428	33.6
Own a mobile phone		
No	537	42.2
Yes	738	57.8

**Note:** Weighted frequencies (*n*) and percentages (%)

### The prevalence of having ever been tested for HIV

**Fig 1:**
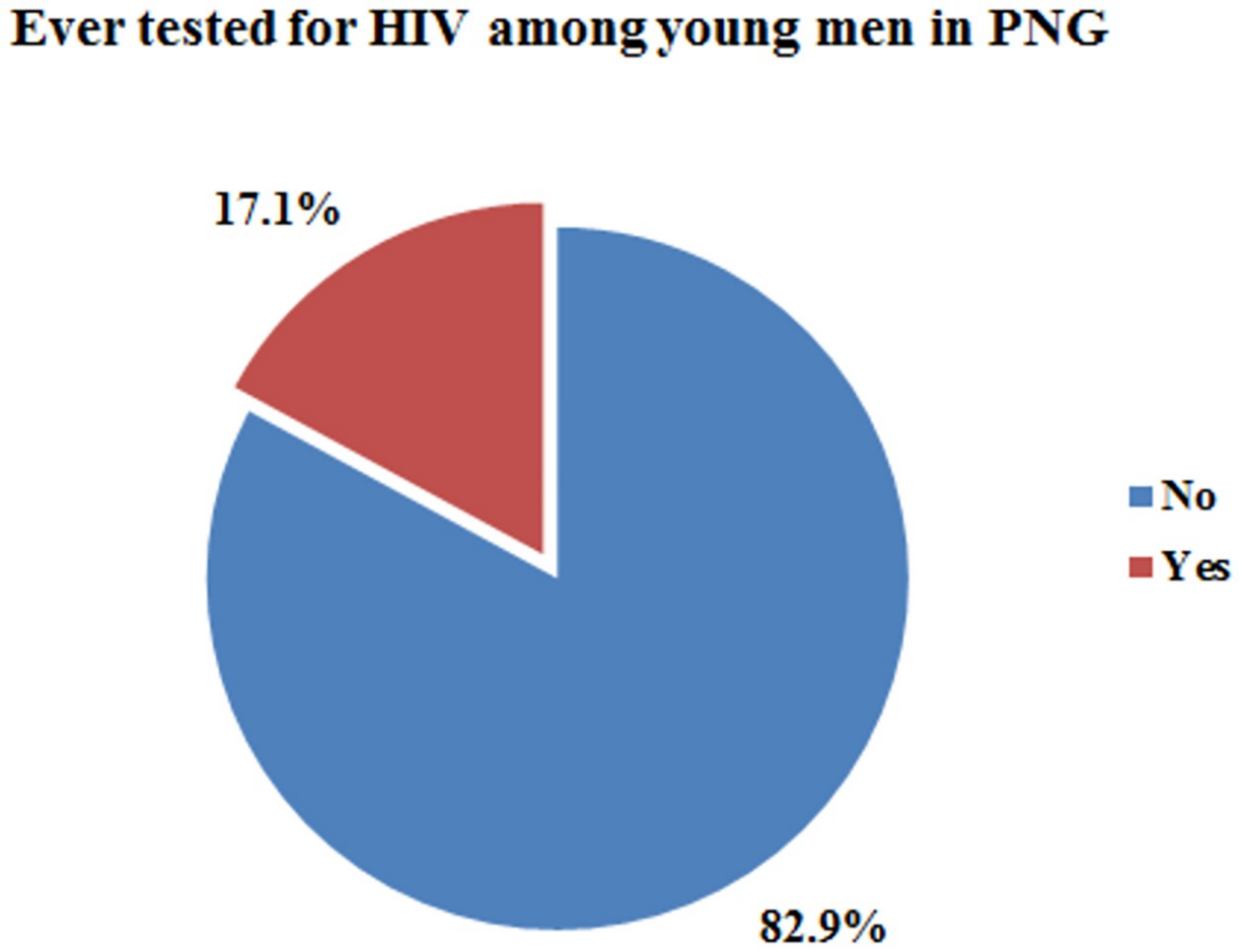
The rates of HIV testing among young men in PNG.

Overall, 17.1% (95% CI: 0.15, 0.19) of the young men had ever been tested for HIV (Fig 1).

### HIV knowledge and sexual risk behaviors

Table 2 shows the HIV-related knowledge and sexual risk behaviors of young men. The vast majority of young men had adequate knowledge that a healthy person can have HIV (90.8%) and consistent condom use during sex can reduce the risk of HIV transmission (80.2%). Most men knew of an HIV testing facility (70.8%). Regarding young men’s sexual risk behaviors, over a quarter were reported to have initiated sex at an early age (<15 years) (28.2%) and inconsistently used condoms during sex (27.7%). The majority of young men had a single sexual partner in the last 12 months (88.7%).

**Table 2:** HIV knowledge and sexual risk behaviors of young men (N = 1,275).

Characteristics	Frequency	
	*n*	%
***HIV Knowledge***		
Ever heard of AIDS		
No	92	7.2
Yes	1,183	92.8
Ever heard of STIs		
No	74	5.8
Yes	1,201	94.2
A healthy person can have HIV (missing, *n* = 326)		
No	88	9.2
Yes	861	90.8
A person can get HIV by sharing food with an infected person (missing, *n* = 298)		
No	822	84.1
Yes	155	15.9
A person can get HIV through witchcraft (missing, *n* = 297)		
No	908	92.8
Yes	70	7.2
Consistent condom use during sex can reduce HIV risk (missing, *n* = 311)		
No	191	19.8
Yes	773	80.2
Know HIV testing facility (missing, *n* = 93)		
No	346	29.2
Yes	836	70.8
***Sexual Risk Behavior***		
Age of sexual debut (years)		
<15	360	28.2
>15	916	71.8
Number of sexual partners, including spouse (last 12 months)		
0–1	1,130	88.7
2 or more	144	11.3
Ever paid for sex (missing, *n* = 49)		
No	1,164	94.9
Yes	63	5.1
Consistent condom use during sex (missing, *n* = 95)		
No	326	27.7
Yes	854	72.3
Had any STIs (last 12 months)^†^		
No	1,211	94.9
Yes	65	5.1

The type of STI was not specified in the dataset.

**Note:** Weighted frequencies (*n*) and percentages (%)

### Bivariate analysis of predictors of HIV testing among young men

Table 3 presents the relationship between sociodemographic characteristics, HIV knowledge, sexual risk behaviors, and HIV testing uptake among young men. Overall, only 17.1% (*n* = 222) of young men have ever been tested for HIV across sociodemographic, HIV knowledge, and sexual risk behavior strata. Having ever been tested for HIV was high among men aged 20–24 years (78.8%), those who were married (66.5%), had attained secondary education (58.1%), were unemployed (53.6%), and lived in rural areas (73%). Similarly, HIV testing remained high among those who read a newspaper or magazine (81.5%), listened to the radio (79.2%), and watched television (53.2%). Regarding HIV knowledge, having ever heard of AIDS and STIs (*p*<0.001), being aware that consistent use of condoms can reduce HIV risk (*p* = 0.011), and knowing an HIV testing facility (*p*<0.001) were significantly associated with HIV testing uptake. Furthermore, there was a statistically significant association between HIV testing and the following sexual risk behavior factors: age of sexual debut (*p* = 0.049), ever paid for sex (*p*<0.001), consistent condom use (*p* = 0.02), and having an STI in the past 12 months (*p*<0.001).

**Table 3:** Bivariate analysis of sociodemographic, HIV knowledge, behavior, and HIV testing (N = 1,275).

Characteristics	Ever been tested for HIV				*P*-value
	No (*n* = 1,053)	%	Yes (*n* = 222)	%	
***Sociodemographics***					
Age (years)					0.002*
15–19	333	31.6	47	21.1	
20–24	720	68.4	175	78.8	
Marital status (missing, *n* = 26)					0.001**
Never married	238	23.0	72	33.5	
Married	796	77.0	143	66.5	
Educational level					0.000**
No formal education	68	6.5	5	2.3	
Primary	490	46.5	75	33.8	
Secondary	450	42.7	129	58.1	
Tertiary	45	4.3	13	5.9	
Employment status					0.005*
Not employed	671	63.7	119	53.6	
Employed	382	36.3	103	46.4	
Wealth Index					0.271
Poorest	96	9.1	24	10.9	
Poorer	94	8.9	25	11.3	
Middle	162	15.4	27	12.2	
Richer	235	22.3	58	26.2	
Richest	466	44.3	87	39.4	
Place of residence					0.000**
Urban	169	16.1	60	27.0	
Rural	883	83.9	162	73.0	
Region					0.000**
Southern	248	23.6	53	23.8	
Highlands	355	33.7	100	44.8	
Momase	332	31.5	41	18.4	
Islands	118	11.2	29	13.0	
Read newspaper/magazine					0.000**
No	328	31.1	41	18.5	
Yes	725	68.9	181	81.5	
Listen to the radio					0.006*
No	315	29.9	46	20.8	
Yes	738	70.1	175	79.2	
Watch television					0.048*
No	570	54.1	104	46.8	
Yes	483	45.9	118	53.2	
Internet access					0.000**
No	730	69.3	117	52.7	
Yes	323	30.7	105	47.3	
Own a mobile phone					0.000**
No	475	45.1	62	28.1	
Yes	578	54.9	159	71.9	
***HIV Knowledge***					
Ever heard of AIDS					0.000**
No	92	8.7	0	0	
Yes	961	91.3	222	100	
Ever heard of STIs					0.000**
No	47	7.0	0	0	
Yes	979	93.0	222	100	
A healthy person can have HIV (missing, *n* = 326)					0.170
No	77	9.9	11	6.5	
Yes	702	90.1	158	93.5	
A person can get HIV by sharing food with an infected person (missing, *n* = 298)					0.248
No	673	83.5	148	87.1	
Yes	133	16.5	22	12.9	
A person can get HIV through witchcraft (missing, *n* = 297)					0.376
No	739	92.5	168	94.4	
Yes	60	7.5	10	5.6	
Consistent condom use during sex can reduce HIV risk (missing, *n* = 311)					0.011*
No	170	21.3	21	12.7	
Yes	627	78.7	145	87.3	
Know HIV testing facility (missing, *n* = 93)					
No	346	36.0	0	0	0.000**
Yes	614	64.0	222	100	
***Sexual Risk Behaviors***					
Age of sexual debut (years)					0.049*
<15	287	27.3	73	32.9	
>15	766	72.7	149	67.1	
Number of sexual partners, including spouse (in the last 12 months)					0.101
0–1	941	89.4	189	85.5	
2 or more	112	10.6	32	14.5	
Ever paid for sex (missing, *n* = 49)					0.000**
No	971	96.7	192	88.1	
Yes	37	3.7	26	11.9	
Consistent condom use during sex (missing, *n* = 95)					0.020*
No	251	26.2	75	33.9	
Yes	707	73.8	146	66.1	
Had any STIs (last 12 months)^†^					0.000**
No	1,010	95.9	200	90.1	
Yes	43	4.1	22	9.9	

*p ≤0.05

**p ≤0.001

^†^The type of STI was not specified in the dataset.

### Multivariable analysis of predictors of HIV testing among young men

Table 4 shows the results of the multivariable analysis examining the association between HIV testing uptake and sociodemographic, HIV-related knowledge, and sexual risk behavior variables. Age, marital status, place of residence, being aware that consistent condom use during sex can reduce HIV risk, ever paid for sex, number of sexual partners, and consistent condom use during sex were statistically significant predictors of HIV testing uptake among young men. In the multivariable analysis, men aged 20–24 years (AOR 1.18, 95% CI: 1.00–2.31), who owned mobile phones (AOR 1.43, 95% CI: 1.00–2.55), who were aware that consistent condom use during sex can reduce HIV risk (AOR 2.18, 95% CI: 1.18–4.04), who had paid for sex (AOR 1.75, 95% CI: 1.01–5.83), and who had two or more sexual partners (AOR 1.37, 95% CI: 1.01–3.14) had increased odds of HIV testing. However, decreased odds of HIV testing uptake were found among men who were never married (AOR 0.51, 95% CI: 0.29–0.88), lived in rural areas (AOR 0.54, 95% CI: 0.32–0.92), and consistently used condoms during sex (AOR 0.59, 95% CI: 0.34–1.01). No significant association was found between age at first sex and HIV testing uptake.

**Table 4:** Multivariable analysis of predictors of HIV testing among young men (N = 1,275).

Characteristics	Ever been tested for HIV				*P*-value
	COR	95% CI	AOR	95% CI	
Age (years)					0.026*
15–19	Ref		Ref		
20–24	1.73	1.09–2.76	1.18	1.00–2.31	
Marital status					0.016*
Never married	0.59	0.39–0.89	0.51	0.29–0.88	
Married	Ref		Ref		
Educational level					0.749
No formal education	0.25	0.05–1.09	0.83	0.13–5.33	
Primary	0.53	0.18–1.58	1.23	0.34–4.49	
Secondary	0.98	0.31–3.09	1.55	0.42–5.69	
Tertiary	Ref		Ref		
Employment status					0.380
Not employed	0.66	0.45–0.97	0.78	0.45–1.36	
Employed	Ref		Ref		
Place of residence					0.023*
Urban	Ref		Ref		
Rural	0.52	0.35–0.77	0.54	0.32–0.92	
Region					0.313
Southern	0.87	0.52–1.46	1.19	1.61–2.32	
Highlands	1.15	0.71–1.86	1.15	0.60–2.18	
Momase	0.49	0.25–1.02	0.61	0.28–1.34	
Islands	Ref		Ref		
Read newspaper/magazine					0.437
No	Ref		Ref		
Yes	1.99	1.32–3.01	1.27	0.69–2.35	
Listen to the radio					0.349
No	Ref		Ref		
Yes	1.61	1.05–2.47	1.38	0.70–2.72	
Watch television					0.291
No	Ref		Ref		
Yes	1.34	0.86–2.09	0.69	0.36–1.36	
Internet access					0.250
No	Ref		Ref		
Yes	2.03	1.26–3.25	1.41	0.79–2.52	
Own a mobile phone					0.026*
No	Ref		Ref		
Yes	2.09	1.38–3.19	1.43	1.00–2.55	
A healthy person can have HIV					0.145
No	Ref		Ref		
Yes	1.56	0.75–3.22	1.95	0.79–4.77	
A person can get HIV by sharing food with an infected person					0.340
No	Ref		Ref		
Yes	0.76	0.42–1.38	0.69	0.33–1/47	
Consistent condom use during sex can reduce HIV risk					0.013*
No	Ref		Ref		
Yes	1.86	1.04–3.33	2.18	1.18–4.04	
Age at first sex					0.509
<15	Ref		Ref		
>15	0.77	0.51–1.17	0.84	0.49–1.43	
Ever paid for sex					0.036*
No	Ref		Ref		
Yes	3.56	1.85–6.84	1.75	1.01–5.83	
Number of sexual partners, including spouse (last 12 months)					0.046*
0–1	Ref		Ref		
2 or more	1.45	0.87–2.40	1.37	1.00–3.14	
Consistent condom use during sex					0.050*
No	Ref		Ref		
Yes	0.69	0.45–1.05	0.59	0.34–1.01	
Had any STIs (last 12 months)					0.639
No	Ref		Ref		
Yes	2.63	1.36–5.09	0.75	0.22–2.54	

*p ≤0.05

COR, crude odds ratio. AOR, adjusted odds ratio. CI, confidence interval. Ref, reference category.

**Note:** Variables such as ever heard of AIDS and STIs had no responses for “ever tested for HIV” and were excluded in the final analysis.

## Discussion

This study assessed the prevalence of HIV testing and determined important sociodemographic factors, HIV-related knowledge, and sexual risk behaviors associated with HIV testing uptake among young men aged 15–24 years using the PNGDHS data. Findings from this study revealed that only 17.1% of young men had ever been tested for HIV, which is lower than that of studies in other countries [38–40]. The dissimilarities could be attributed to differences in HIV knowledge and awareness and the availability and accessibility of HIV testing services in these countries. There is evidence that young men’s low uptake of HIV testing may be attributed to a lack of access to HIV testing services and loss of trust in these services [7, 41]. In addition, fear of stigmatization, fear of being diagnosed positive for HIV, the perceived risk of sexual exposure, poor attitudes by healthcare professionals, and the perceived psychological burden have been found to impede HIV testing uptake [7]. In PNG, HIV testing services have been more focused on the prevention of parent-to-child transmission [42–44] and key populations (e.g., sex workers) [45, 46], while such progress is yet to be achieved with high-risk young populations. Given the importance of early HIV diagnosis and linking access to treatment and care, age-specific policy development is required to increase HIV testing uptake among this priority group.

Consistent with previous studies [47–49], this study indicated that demographic factors, such as age, marital status, and place of residence were significant predictors of HIV testing uptake among young men. In this study, HIV testing uptake tends to be significantly higher with increasing age, with men aged 20–24 years being 1.18 times more likely to be tested than adolescent men (aged 15–19 years). A plausible explanation could be that they have a lower self-perceived likelihood of HIV compared to those of increased age, who are more likely to be sexually active and knowledgeable about HIV and its related risks [47, 49, 50]. It is also possible that they are more likely to have married and have more lifetime exposure to HIV testing services, such as during antenatal care [47, 51]. Health education and awareness, including school-based interventions to increase HIV knowledge and risk perceptions, are feasible options that could promote positive sexual risk behaviors and increase HIV testing uptake among young men.

There was a negative association between marital status and HIV testing uptake. The odds of HIV testing remained lower among unmarried young men than those who were married. The result corroborated recent studies in Sub-Saharan Africa [47, 52, 53]. One possible explanation for this could be that young men frequently encounter challenges accessing available HIV testing services, are dissatisfied with the services provided, and have confidentiality issues [7, 49]. Also, another reason could be that young men have a low-risk perception and are unaware of the physical signs of HIV and its adverse consequences [54]. Evidence has shown that young men with low-risk perceptions who do not have access to HIV testing services are less likely to have received an HIV test in their lifetime [55, 56]. Youth-friendly health services can help close the HIV testing gap among young men by addressing access barriers, providing a more accessible, equitable, and effective social environment, and linking to HIV treatment and care.

The results further revealed that the place of residence was negatively associated with HIV testing. Young men who were from rural areas were less likely to be tested for HIV, consistent with previous studies elsewhere [53, 57]. The possible explanation for this could be due to the availability and accessibility of HIV testing services in urban areas rather than in rural areas, where young men would have fewer opportunities to be tested for HIV. This disparity may also reflect a lack of HIV services or HIV-trained health workers, and inequitable outreach programs to reach young men in rural areas, as previously reported [58, 59]. While prioritizing areas that require tailored and youth-specific interventions to improve HIV testing access and uptake, there is a need to further expand HIV services to rural areas. Mobile and rural health outreach programs are critical for reaching young men lacking health care access and increasing HIV testing services.

In agreement with studies in Kenya and Ethiopia [60, 61], the current study found that mobile phone ownership among young men increases the uptake of HIV testing compared to non-mobile owners. Mobile phone interventions in healthcare (mHealth) have shown success in improving access to care and treatment, especially in settings where health disparities are more pronounced [62, 63]. Evidence is emerging that the use of mobile technologies remains an effective strategy to reach young people improve access to treatment and retention in care and provide information on HIV services and prevention interventions, including risk reduction [60, 64, 65]. Furthermore, mobile phones have been shown to address barriers of provider prejudice, stigmatization, discrimination, fear of refusal, lack of privacy, and confidentiality [66, 67]. Using mHealth technology provides a potentially promising implementation strategy for interventions to remedy disparities and barriers to HIV testing and the care continuum among young men. The study underscores the significance of preliminary, formative research on population disparities in accessibility, effectiveness, willingness, and the use of mobile phones in HIV prevention programs.

In this study, young men who knew that consistent condom use during sex could reduce HIV risk were 2.18 times more likely to be tested for HIV than their counterparts. The results were similar to previous studies in Ethiopia and Iran [68, 69], indicating that having a better knowledge of condom efficacy and effectiveness and sexual modes of HIV transmission were associated with condom use. Additionally, this study established that young men who consistently use condoms during sex were less likely to be tested for HIV. This is consistent with evidence suggesting that increased knowledge of HIV and risk perceptions were associated with a positive attitude toward condom use [68, 69]. It is possible that discussion of HIV with a sexual partner, knowing the partner’s HIV status, and being in a steady relationship influenced the consistent use of condoms [70, 71]. Regular health education and information dissemination on condom self-efficacy and risk reduction are crucial for preventing HIV infection among this priority group.

Regarding sexual risk behaviors, young men who had ever paid for sex were more likely to be tested for HIV than their counterparts. These findings are consistent with previous studies elsewhere [72, 73], which showed that young men who engage in risky sexual behaviors such as sex work and inconsistently use condoms appropriately perceive their risks of HIV acquisition and transmission and, as a result, undertake HIV testing. The fact that they engage in such risky behaviors may imply that their overall risk perception differs from that of those who do not engage in risky behaviors [74]. Strategies for young men who paid for sex should not only concentrate on HIV testing and treatment approaches, but also on the social contexts in which they engage in transacting sex. Similarly, having multiple sexual partners was significantly associated with HIV testing uptake. This finding corroborates a study in Canada [75] that found an association between HIV testing and having concurrent and multiple sexual partners among men. Having concurrent and multiple sexual partners and risky sexual behaviors are predisposed to HIV and other STIs. Evidence shows that young people who have multiple sex partners and are knowledgeable about HIV and its risks are more likely to utilize HIV testing services [75, 76]. The positive association between having multiple partners and the uptake of HIV testing implies that there is a need for developing targeted programs for behavior change and risk reduction for young people in this context.

Contrary to other studies [7, 47], no association between the uptake of HIV testing and educational level, employment status, wealth index, mass media exposure, age of sexual debut, or having an STI was found in this study. However, this study has provided useful findings that have the potential to inform the strengthening of current HIV prevention programs to target young men in PNG. Improving HIV testing uptake for young people will play a vital role in reducing the risk of acquiring HIV among young men, hence optimizing their health and well-being [54]. Targeted and coordinated programs aimed at increasing HIV testing services among this priority group remain imperative to achieve the national targets, and should not be neglected.

### Study strengths and limitations

The study is based on nationwide population data with a nationally representative sample. Also, appropriate estimation adjustments, such as weighting, were employed and this has helped to strengthen the validity and generalizability of the study findings. However, the study has limitations, and findings should be interpreted cautiously. Given that the data are from a cross-sectional study, it is impossible to prove the temporal relationship. Since the study’s outcome depended on self-reporting, social desirability bias is highly probable, which might have led to under-reporting by the participants. In addition, the dataset did not indicate whether young men were in same-sex relationships (such as men who have sex with men) or not, which could have influenced the uptake of HIV testing.

## Conclusion

Findings from this study showed that one in six young men had ever tested for HIV in PNG, which is well below the national HIV/STI targets. Men aged 20–24 years who owned mobile phones, were aware that consistent condom use reduces HIV risks, had ever paid for sex, and had multiple sexual partners were significant predictors of HIV testing. To increase HIV testing uptake among young men, it is crucial to implement targeted and comprehensive youth-friendly HIV/STI education and sensitization programs and enable more accessible and affordable HIV testing services. Moreover, concerted efforts for outreach and community-based testing programs, especially in rural and prioritized areas where HIV testing services are inaccessible, are feasible options for young men in PNG.

## Supporting information

S1 Checklist STROBE checklist. (DOCX)

S1 Dataset HIV testing-young men dataset. (XLSX)

## References

[1] Assefa Y, Gilks CF. Ending the epidemic of HIV/AIDS by 2030: Will there be an endgame to HIV, or an endemic HIV requiring an integrated health systems response in many countries? International Journal of Infectious Diseases. 2020;100: 273–277. doi: 10.1016/j.ijid.2020.09.011 32920236

[2] UNAIDS. The path that ends AIDS: UNAIDS Global AIDS Update 2023. Geneva, Switzerland; 2023. https://thepath.unaids.org/

[3] UNAIDS. Understanding Fast-Track: Accelerating action to end the AIDS Epidemic by 2030. Geneva, Switzerland; 2015. https://www.unaids.org/sites/default/files/media_asset/201506_JC2743_Understanding_FastTrack_en.pdf

[4] Frescura L, Godfrey-Faussett P, Feizzadeh A. A, El-Sadr W, Syarif O, Ghys PD, et al. Achieving the 95 95 95 targets for all: A pathway to ending AIDS. PLOS ONE. 2022;17: e0272405. doi: 10.1371/journal.pone.0272405 35925943 PMC9352102

[5] Delavande A, Kohler H-P. The Impact of HIV Testing on Subjective Expectations and Risky Behavior in Malawi. Demography. 2012;49: 1011–1036. doi: 10.1007/s13524-012-0119-7 22744765 PMC3906596

[6] Scott-Sheldon LA, Carey MP, Carey KB, Cain D, Simbayi LC, Mehlomakhulu V, et al. HIV testing is associated with increased knowledge and reductions in sexual risk behaviours among men in Cape Town, South Africa. African Journal of AIDS Research. 2013;12: 195–201. doi: 10.2989/16085906.2013.863219 25871481 PMC4520431

[7] Ajayi AI, Awopegba OE, Adeagbo OA, Ushie BA. Low coverage of HIV testing among adolescents and young adults in Nigeria: Implication for achieving the UNAIDS first 95. PLOS ONE. 2020;15: e0233368. doi: 10.1371/journal.pone.0233368 32428005 PMC7237011

[8] Mwaba K, Mannell J, Burgess R, Sherr L. Uptake of HIV testing among 15–19-year-old adolescents in Zambia. AIDS Care. 2020;32: 183–192. doi: 10.1080/09540121.2020.1739214 32169008

[9] Fauk NK, Sukmawati AS, Berek PAL, Ernawati, Kristanti E, Wardojo SSI, et al. Barriers to HIV testing among male clients of female sex workers in Indonesia. International Journal for Equity in Health. 2018;17: 68. doi: 10.1186/s12939-018-0782-4 29848324 PMC5977459

[10] Hlongwa M, Mashamba-Thompson T, Makhunga S, Hlongwana K. Barriers to HIV testing uptake among men in sub-Saharan Africa: a scoping review. African Journal of AIDS Research. 2020;19: 13–23. doi: 10.2989/16085906.2020.1725071 32174231

[11] Tetteh JK, Frimpong JB, Budu E, Adu C, Mohammed A, Ahinkorah BO, et al. Comprehensive HIV/AIDS knowledge and HIV testing among men in sub-Saharan Africa: a multilevel modelling. Journal of Biosocial Science. 2021/11/05 ed. 2022;54: 975–990. doi: 10.1017/S0021932021000560 34736542

[12] Caltabiano M, Castiglioni M, De-Rose A. Changes in the sexual behaviour of young people: introduction. Genus. 2020;76: 38. doi: 10.1186/s41118-020-00107-1

[13] Hittner JB, Owens EC, Swickert RJ. Influence of Social Settings on Risky Sexual Behavior. SAGE Open. 2016;6: 2158244016629187. doi: 10.1177/2158244016629187

[14] Cho H-S, Yang Y. Relationship Between Alcohol Consumption and Risky Sexual Behaviors Among Adolescents and Young Adults: A Meta-Analysis. International Journal of Public Health. 2023;68. doi: 10.3389/ijph.2023.1605669 37153699 PMC10154531

[15] Harris B, McCredie MN, Truong T, Regan T, Thompson CG, Leach W, et al. Relations Between Adolescent Sensation Seeking and Risky Sexual Behaviors Across Sex, Race, and Age: A Meta-Analysis. Archives of Sexual Behavior. 2023;52: 191–204. doi: 10.1007/s10508-022-02384-7 36121585

[16] Manu A, Ogum-Alangea D, Azilaku JC, Anaba EA, Torpey K. Risky sexual behaviours and HIV testing among young people in Ghana: evidence from the 2017/2018 Multiple Indicator Cluster Survey. Reproductive Health. 2022;19: 125. doi: 10.1186/s12978-022-01439-1 35643502 PMC9148450

[17] Bolarinwa OA, Ajayi KV, Sah RK. Association between knowledge of Human Immunodeficiency Virus transmission and consistent condom use among sexually active men in Nigeria: An analysis of 2018 Nigeria Demographic Health Survey. Jilo GK, editor. PLOS Glob Public Health. 2022;2: e0000223. doi: 10.1371/journal.pgph.0000223 36962299 PMC10021623

[18] Wilcken A, Miiro-Nakayima F, Hizaamu RN, Keil T, Balaba-Byansi D. Male circumcision for HIV prevention—a cross-sectional study on awareness among young people and adults in rural Uganda. BMC Public Health. 2010;10: 209. doi: 10.1186/1471-2458-10-209 20420701 PMC2880292

[19] Bendera A, Nakamura K, Seino K, Al-Sobaihi S. Factors Associated with Low Uptake of Medical Male Circumcision Among Adolescent Boys in Tanzania: A Multinomial Logistic Regression Modeling. HIV/AIDS—Research and Palliative Care. 2022;14: 565–575. doi: 10.2147/HIV.S387380 36571074 PMC9785118

[20] Folayan MO, Sam-Agudu NA, Harrison A. Exploring the why: risk factors for HIV and barriers to sexual and reproductive health service access among adolescents in Nigeria. BMC Health Services Research. 2022;22: 1198. doi: 10.1186/s12913-022-08551-9 36151543 PMC9508705

[21] Alexis Sentís Mario Martin-Sanchez, Arando Maider, Vall Martí, María Jesus Barbera Inma Ocaña, et al. Sexually transmitted infections in young people and factors associated with HIV coinfection: an observational study in a large city. BMJ Open. 2019;9: e027245. doi: 10.1136/bmjopen-2018-027245 31061051 PMC6502227

[22] Pettifor A, Bekker L-G, Hosek S, DiClemente R, Rosenberg M, Bull SS, et al. Preventing HIV among young people: research priorities for the future. J Acquir Immune Defic Syndr. 2013;63 Suppl 2: S155–S160. doi: 10.1097/QAI.0b013e31829871fb 23764629 PMC3746811

[23] Pettifor A, Stoner M, Pike C, Bekker L-G. Adolescent lives matter: preventing HIV in adolescents. Curr Opin HIV AIDS. 2018;13: 265–273. doi: 10.1097/COH.0000000000000453 29528850 PMC5902132

[24] Hakim AJ, Coy K, Badman SG, Willie B, Narokobi R, Gabuzzi J, et al. One size does not fit all: HIV prevalence and correlates of risk for men who have sex with men, transgender women in multiple cities in Papua New Guinea. BMC Public Health. 2019;19: 623. doi: 10.1186/s12889-019-6942-7 31117978 PMC6532262

[25] Kelly-Hanku A, Willie B, Weikum D, Boli Neo R, Kupul M, Coy K. Kauntim mi tu: Multi-site summary report from the key population integrated bio-behavioural survey, Papua New Guinea. Goroka: Papua New Guinea Institute of Medical Research and Kirby Institute, UNSW Sydney. 2018. https://www.kirby.unsw.edu.au/research/reports/kauntim-mi-tu-port-moresby-2017

[26] UNAIDS. Country facts—Papua New Guinea 2022. UNAIDS; 2022. https://www.unaids.org/en/regionscountries/countries/papuanewguinea

[27] Grundy J, Dakulala P, Wai K, Maalsen A, Whittaker M. Independent State of Papua New Guinea Health System Review. World Health Organization. Regional Office for South-East Asia; 2019. https://iris.who.int/handle/10665/280088

[28] Maviso M, Kalembo FW. Prevalence and determinants of not testing for HIV among young adult women in Papua New Guinea: findings from the Demographic and Health Survey, 2016–2018. BMJ Open. 2024;14: e075424. doi: 10.1136/bmjopen-2023-075424 38453195 PMC10921496

[29] Worth H. HIV Prevention in Papua New Guinea: Is It Working or Not? World Journal of AIDS. 2012;2: 117.

[30] Baigry MI, Ray R, Lindsay D, Kelly-Hanku A, Redman-MacLaren M. Barriers and enablers to young people accessing sexual and reproductive health services in Pacific Island Countries and Territories: A scoping review. PLOS ONE. 2023;18: e0280667. doi: 10.1371/journal.pone.0280667 36701390 PMC9879431

[31] Gray N, Azzopardi P, Kennedy E, Willersdorf E, Creati M. Improving Adolescent Reproductive Health in Asia and the Pacific: Do We Have the Data? A Review of DHS and MICS Surveys in Nine Countries. Asia Pac J Public Health. 2013;25: 134–144. doi: 10.1177/1010539511417423 21807622

[32] Kelly A, Worth H, Akuani F, Kepa B, Kupul M, Walizopa L, et al. Gendered talk about sex, sexual relationships and HIV among young people in Papua New Guinea. Culture, Health & Sexuality. 2010;12: 221–232. doi: 10.1080/13691050903181107 19813120

[33] Kuzma J, Himata R. A pilot study of the knowledge, behaviour and attitudes connected with HIV/AIDS among youth in PNG. Contemporary PNG Studies. 2005;3: 1–12.

[34] Yakam LT. Factors associated with alcohol consumption among students in Divine Word University, Madang Province, Papua New Guinea. Contemporary PNG Studies. 2021;34: 17–27.

[35] National Statistical Office (NSO). National Population and Housing Census 2011. Port Moresby, Papua New Guinea: National Statistical Office; 2015. https://png-data.sprep.org/resource/papua-new-guinea-2011-national-report

[36] National Statistical Office (NSO) [Papua New Guinea], ICF. Papua New Guinea Demographic and Health Survey 2016–18. Port Moresby, Papua New Guinea, and Rockville, Maryland, USA: NSO and ICF; 2019. https://www.dhsprogram.com/pubs/pdf/FR364/FR364.pdf

[37] Zhang Z. Model building strategy for logistic regression: purposeful selection. Annals of Translational Medicine; Vol 4, No 6 (March 25, 2016): Annals of Translational Medicine (Toward Precision Medicine in Neurological Diseases). 2016 [cited 31 Dec 2015]. 10.21037/atm.2016.02.15 27127764 PMC4828741

[38] Asaolu IO, Gunn JK, Center KE, Koss MP, Iwelunmor JI, Ehiri JE. Predictors of HIV Testing among Youth in Sub-Saharan Africa: A Cross-Sectional Study. PLOS ONE. 2016;11: e0164052. doi: 10.1371/journal.pone.0164052 27706252 PMC5051677

[39] Ijaiya MA, Anibi A, Abubakar MM, Obanubi C, Anjorin S, Uthman OA. A multilevel analysis of the determinants of HIV testing among men in Sub-Saharan Africa: Evidence from Demographic and Health Surveys across 10 African countries. PLOS Global Public Health. 2024;4: e0003159. doi: 10.1371/journal.pgph.0003159 38696392 PMC11065312

[40] Olakunde BO, Adeyinka DA, Olawepo JO, Pharr JR. HIV testing among men in Nigeria: a comparative analysis between young people and adults. AIDS Care. 2020;32: 155–162. doi: 10.1080/09540121.2019.1622642 31137949

[41] Aluzimbi G, Lubwama G, Muyonga M, Hladik W. HIV Testing and Risk Perceptions: A Qualitative Analysis of Secondary School Students in Kampala, Uganda. J Public Health Afr. 2017;8: 577. 28878868 10.4081/jphia.2017.577 PMC5575453

[42] Carmone A, Bomai K, Bongi W, Frank TD, Dalepa H, Loifa B, et al. Partner testing, linkage to care, and HIV-free survival in a program to prevent parent-to-child transmission of HIV in the Highlands of Papua New Guinea. Global Health Action. 2014;7: 24995. doi: 10.3402/gha.v7.24995 25172429 PMC4149744

[43] Badman SG, Vallely LM, Toliman P, Kariwiga G, Lote B, Pomat W, et al. A novel point-of-care testing strategy for sexually transmitted infections among pregnant women in high-burden settings: results of a feasibility study in Papua New Guinea. BMC Infectious Diseases. 2016;16: 250. doi: 10.1186/s12879-016-1573-4 27268218 PMC4895793

[44] Saweri OP, Batura N, Pulford J, Khan MM, Hou X, Pomat WS, et al. Investigating health service availability and readiness for antenatal testing and treatment for HIV and syphilis in Papua New Guinea. BMC Pregnancy and Childbirth. 2022;22: 780. doi: 10.1186/s12884-022-05097-w 36261790 PMC9580192

[45] Hakim AJ, Badman SG, Weikum D, Amos A, Willie B, Narokobi R, et al. Considerable distance to reach 90-90-90 targets among female sex workers, men who have sex with men and transgender women in Port Moresby, Papua New Guinea: findings from a cross-sectional respondent-driven sampling survey. Sex Transm Infect. 2020;96: 143. 10.1136/sextrans-2019-053961 31182653 PMC8678575

[46] Mitchell E, Hakim A, Nosi S, Kupul M, Boli-Neo R, Aeno H, et al. A socio-ecological analysis of factors influencing HIV treatment initiation and adherence among key populations in Papua New Guinea. BMC Public Health. 2021;21: 2003. doi: 10.1186/s12889-021-12077-w 34736447 PMC8567601

[47] Sonko I, Chung M-H, Hou W-H, Chen W-T, Chang P-C. Predictors of HIV testing among youth aged 15–24 years in The Gambia. PLOS ONE. 2022;17: e0263720. doi: 10.1371/journal.pone.0263720 35180256 PMC8856544

[48] Ajiboye AS, Eshetu F, Lulseged S, Getaneh Y, Tademe N, Kifle T, et al. Predictors of HIV testing among youth 15–24 years in urban Ethiopia, 2017–2018 Ethiopia population-based HIV impact assessment. PLOS ONE. 2023;18: e0265710. doi: 10.1371/journal.pone.0265710 37467301 PMC10355388

[49] Alem AZ, Liyew AM, Guadie HA. Spatial pattern and associated factors of HIV testing and counselling among youths (15–24 years) in Ethiopia. BMC Public Health. 2021;21: 644. doi: 10.1186/s12889-021-10677-0 33794831 PMC8017837

[50] Wang Y, Kinsler JJ, Kiwuwa-Muyingo S. Factors associated with HIV testing among youth in Tanzania based on the 2016–2017 Tanzania HIV Impact Survey (THIS). PLOS Global Public Health. 2022;2: e0000536. doi: 10.1371/journal.pgph.0000536 36589732 PMC9799957

[51] Kalibbala D, Mpungu SK, Ssuna B, Muzeyi W, Mberesero H, Semitala FC, et al. Determinants of testing for HIV among young people in Uganda. A nested, explanatory-sequential study. PLOS Global Public Health. 2022;2: e0000870. doi: 10.1371/journal.pgph.0000870 36962841 PMC10022384

[52] Nshimirimana C, Vuylsteke B, Smekens T, Benova L. HIV testing uptake and determinants among adolescents and young people in Burundi: a cross-sectional analysis of the Demographic and Health Survey 2016–2017. BMJ Open. 2022;12: e064052. 10.1136/bmjopen-2022-064052 36229150 PMC9562752

[53] Adugna DG, Worku MG. HIV testing and associated factors among men (15–64 years) in Eastern Africa: a multilevel analysis using the recent demographic and health survey. BMC Public Health. 2022;22: 2170. doi: 10.1186/s12889-022-14588-6 36434555 PMC9701050

[54] Musonda E, Phiri M, Shasha L, Bwalya C, Musemangezhi S, Ishimwe SMC, et al. Prevalence of HIV testing uptake among the never-married young men (15–24) in sub-Saharan Africa: An analysis of demographic and health survey data (2015–2020). PLOS ONE. 2023;18: e0292182. doi: 10.1371/journal.pone.0292182 37796957 PMC10553359

[55] Jung MS, Dlamini NS, Cui X, Cha K. Prevalence of HIV testing and associated factors among young adolescents in Eswatini: a secondary data analysis. BMC Pediatrics. 2022;22: 659. doi: 10.1186/s12887-022-03698-0 36376807 PMC9661805

[56] Muravha T, Hoffmann CJ, Botha C, Maruma W, Charalambous S, Chetty-Makkan CM. Exploring perceptions of low risk behaviour and drivers to test for HIV among South African youth. PLOS ONE. 2021;16: e0245542. doi: 10.1371/journal.pone.0245542 33481878 PMC7822253

[57] Mabaso M, Maseko G, Sewpaul R, Naidoo I, Jooste S, Takatshana S, et al. Trends and correlates of HIV prevalence among adolescents in South Africa: evidence from the 2008, 2012 and 2017 South African National HIV Prevalence, Incidence and Behaviour surveys. AIDS Research and Therapy. 2021;18: 97. doi: 10.1186/s12981-021-00422-3 34906170 PMC8670218

[58] Sanga ES, Mukumbang FC, Mushi AK, Lerebo W, Zarowsky C. Understanding factors influencing linkage to HIV care in a rural setting, Mbeya, Tanzania: qualitative findings of a mixed methods study. BMC Public Health. 2019;19: 383. doi: 10.1186/s12889-019-6691-7 30953503 PMC6451278

[59] Pellowski JA. Barriers to care for rural people living with HIV: a review of domestic research and health care models. J Assoc Nurses AIDS Care. 2013;24: 422–437. doi: 10.1016/j.jana.2012.08.007 23352771 PMC3640620

[60] Jennings Mayo-Wilson L, Kang B-A, Mathai M, Mak’anyengo MO, Ssewamala FM. Mobile phone access, willingness, and usage for HIV-related services among young adults living in informal urban settlements in Kenya: A cross-sectional analysis. International Journal of Medical Informatics. 2022;161: 104728. doi: 10.1016/j.ijmedinf.2022.104728 35228007 PMC8940651

[61] Nigatu M, Kabeta T, Taye A, Belina M. HIV voluntary counseling and testing uptake and associated factors among Ethiopian youths: evidence from the 2016 EDHS using multilevel modeling. BMC Infectious Diseases. 2021;21: 334. doi: 10.1186/s12879-021-06021-x 33836650 PMC8033676

[62] Hall CS, Fottrell E, Wilkinson S, Byass P. Assessing the impact of mHealth interventions in low- and middle-income countries–what has been shown to work? Global Health Action. 2014;7: 25606. doi: 10.3402/gha.v7.25606 25361730 PMC4216389

[63] Nwaozuru U, Obiezu-Umeh C, Shato T, Uzoaru F, Mason S, Carter V, et al. Mobile health interventions for HIV/STI prevention among youth in low- and middle-income countries (LMICs): a systematic review of studies reporting implementation outcomes. Implement Sci Commun. 2021;2: 126–126. doi: 10.1186/s43058-021-00230-w 34742357 PMC8572487

[64] Garg PR, Uppal L, Mehra S, Mehra D. Mobile Health App for Self-Learning on HIV Prevention Knowledge and Services Among a Young Indonesian Key Population: Cohort Study. JMIR Mhealth Uhealth. 2020;8: e17646–e17646. doi: 10.2196/17646 32896831 PMC7509613

[65] Ippoliti NB, L’Engle K. Meet us on the phone: mobile phone programs for adolescent sexual and reproductive health in low-to-middle income countries. Reproductive Health. 2017;14: 11. doi: 10.1186/s12978-016-0276-z 28095855 PMC5240300

[66] Mulawa MI, Rosengren AL, Amico KR, Hightow-Weidman LB, Muessig KE. mHealth to reduce HIV-related stigma among youth in the United States: a scoping review. Mhealth. 2021;7: 35. 10.21037/mhealth-20-68 33898604 PMC8063007

[67] Feroz AS, Ali NA, Khoja A, Asad A, Saleem S. Using mobile phones to improve young people sexual and reproductive health in low and middle-income countries: a systematic review to identify barriers, facilitators, and range of mHealth solutions. Reprod Health. 2021;18: 9–9. doi: 10.1186/s12978-020-01059-7 33453723 PMC7811742

[68] Hosseini Hooshyar S, Karamouzian M, Mirzazadeh A, Haghdoost AA, Sharifi H, Shokoohi M. Condom Use and its Associated Factors Among Iranian Youth: Results From a Population-Based Study. Int J Health Policy Manag. 2018;7: 1007–1014. doi: 10.15171/ijhpm.2018.65 30624874 PMC6326636

[69] Yosef T, Nigussie T. Behavioral Profiles and Attitude toward Condom Use among College Students in Southwest Ethiopia. BioMed Research International. 2020;2020: 1–6. doi: 10.1155/2020/9582139 33029533 PMC7532416

[70] Ajayi AI, Ismail KO, Akpan W. Factors associated with consistent condom use: a cross-sectional survey of two Nigerian universities. BMC Public Health. 2019;19: 1207. doi: 10.1186/s12889-019-7543-1 31477068 PMC6719351

[71] Ajayi OA, Ogunsola OO, Akinro Y, Adamu-Oyegun S, Wudiri K, Ojo TO, et al. Consistent condom use and associated factors among HIV positive women of reproductive age on anti-retroviral treatment in Ogun State, Nigeria. Pan Afr Med J. 2022;43: 101. doi: 10.11604/pamj.2022.43.101.32806 36699975 PMC9834802

[72] Hodgins C, Stannah J, Kuchukhidze S, Zembe L, Eaton JW, Boily M-C, et al. Population sizes, HIV prevalence, and HIV prevention among men who paid for sex in sub-Saharan Africa (2000–2020): A meta-analysis of 87 population-based surveys. PLOS Medicine. 2022;19: e1003861. doi: 10.1371/journal.pmed.1003861 35077459 PMC8789156

[73] Gebresilassie F, Ayele B, Hadgu T, Gebretnsae H, Negash D, Demoz Ghebremdhin K, et al. Predictors of Condom Use Among Youth of the Rural Tigray, Northern Ethiopia: Community-Based Cross-Sectional Study. HIV/AIDS—Research and Palliative Care. 2023;15: 377–385. doi: 10.2147/HIV.S412337 37377455 PMC10292206

[74] Dizechi S, Brody C, Tuot S, Chhea C, Saphonn V, Yung K, et al. Youth paying for sex: what are the associated factors? Findings from a cross-sectional study in Cambodia. BMC Public Health. 2018;18: 113. doi: 10.1186/s12889-017-4999-8 29310630 PMC5759259

[75] Luginaah NA, Konkor I, Lawson ES, Mkandawire P, Husbands W, Omorodion F, et al. Concurrent sexual partnerships and HIV testing among heterosexual Black men in Ontario, Canada: findings from the weSpeak study. Ethn Health. 2022;27: 1825–1840. doi: 10.1080/13557858.2021.1976395 34494926

[76] N Wilson Chialepeh, A Sathiyasusuman. Associated Risk Factors of STIs and Multiple Sexual Relationships among Youths in Malawi. PLOS ONE. 2015;10: e0134286. doi: 10.1371/journal.pone.0134286 26248328 PMC4527764

